# Yeast Cell Wall Compounds on The Formation of Fermentation Products and Fecal Microbiota in Cats: An In Vivo and In Vitro Approach

**DOI:** 10.3390/ani13040637

**Published:** 2023-02-11

**Authors:** Fernando González, Amanda Carelli, Alina Komarcheuski, Mayara Uana, Rodolpho Martin do Prado, Diogo Rossoni, Márcia Gomes, Ricardo Vasconcellos

**Affiliations:** 1 Department of Internal Medicine, College of Veterinary Medicine and Animal Science, University of São Paulo (USP)—São Paulo, Av. Prof. Dr. Orlando Marques de Paiva, 87, São Paulo 13690-970, Brazil; 2 Department of Animal Science, State University of Maringá, Maringá, Av. Colombo, 5790, Maringá 87020-900, Brazil

**Keywords:** beta-glucan, butyrate, feline, lactate, mannan oligosaccharide, microbiota

## Abstract

**Simple Summary:**

This study assessed the in vivo and in vitro fermentative responses and fecal microbiota in cats fed diets supplemented with a blend of yeast cell wall compounds (YCWs). Results were better expressed in vitro; however, both experiments showed that a low dose of YCWs could modulate the metabolism and fecal microbiota traits in cats. This effect was observed via changes in fermentation product concentrations and suggested that using YCWs could potentially contribute to the intestinal health of adult cats fed extruded diets.

**Abstract:**

The effects of yeast cell wall compounds (YCWs) being added to cat food on hindgut fermentation metabolites and fecal microbiota were assessed in in vivo Experiment 1 (Exp. 1) and in vitro Experiments 2 and 3 (Exp. 2 and 3). In Exp. 1, the cats’ diets were supplemented with two dietary concentrations (46.2 and 92.4 ppm) of YCWs (YCW-15 and YCW-30, respectively), and a negative control diet with no compound in three groups (six cats per group) was used to assess the fecal score, pH, digestibility, fermentation products, and microbiota. In Exp. 2, feces from the cats that were not supplemented with YCWs (control) were used as an inoculum. A blend of pectin, amino acids, and cellulose was used as a substrate, and the YCW compound was added at two levels (5 and 10 mg). In Exp. 3, feces from cats fed YCWs were used as an inoculum to test three different substrates (pectin, amino acids, and cellulose). In Exp. 2 and 3, the gas production, pH, and fermentation products (ammonia, SCFAs, and BCFAs) were assessed. YCW-30 resulted in a higher digestibility coefficient of the crude protein, organic matter (OM) (*p* < 0.05), and energy of the diet (*p* < 0.10). Regarding the fermentation products, YCW-15 showed a trend toward higher concentrations of propionate, acetate, lactate, ammonia, isobutyrate, and valerate, while YCW-30 showed a trend (*p* < 0.10) toward higher levels of butyrate and pH values. The bacteroidia class and the genus Prevotella were increased by using YCW-30 and the control. At the gender level, decreased (*p* < 0.01) Megasphaera was observed with YCW inclusion. The microbiota differed (*p* < 0.01) among the groups in their Shannon indexes. For beta diversity, YCW-30 showed higher indexes (*p* = 0.008) than the control. The microbiota metabolic profile differed in the pathway CENTFERM-PWY; it was more expressed in YCW-30 compared to the control. In Exp. 2, the YCWs showed a higher ratio (*p* = 0.006) of the fermentation products in the treatments with additives with a trend towards a high dose of the additive (10 mg). In Exp. 3, the effects of the substrates (*p* < 0.001), but not of the YCWs, on the fermentation products were observed, perhaps due to the low dietary concentrations we used. However, the marked responses of the fermentation products to the substrates validated the methodology. We could conclude that the YCWs, even at low dietary concentrations, affected fecal SCFA production, reduced the fecal pH, and modulated the fecal microbiota in the cats. These responses were more pronounced under in vitro conditions.

## 1. Introduction

Domestic cats are evolutionarily strict carnivores. However, despite having a short colon and a nonfunctional cecum, they have a considerable fermentative capacity [1]. Thus, feeding cats nutrients that possess prebiotic activity is also important to their intestinal microbiota modulation and, hence, to their health [2,3,4,5]. Yeast *(Saccharomyces cerevisiae*) derivatives, such as beta-glucans and mannan oligosaccharides (MOSs), are widely used as prebiotics in the pet food industry due to their large-scale production [6]. When proper dietary doses are used, yeast derivatives can benefit the intestinal environment and fecal quality; for instance, the yeast derivatives’ effects include a lower intestinal pH, greater microbiota diversity, reduced protein fermentation products, an improved inflammatory response, and improved intestinal health markers [2,7].

Microbiota and dietary substrates can affect the intestinal concentration of fermentation products. Indeed, studies have shown that the diet can change this composition in the host and that either health-promoting or undesirable compounds may be produced [2,4,5,8]. One of the main benefits of intestinal microbiota modulation is the increase in short-chain fatty acid (SCFA) synthesis due to microbial carbohydrate degradation (fiber, starch, and sugars) [9,10,11]. Microbial protein degradation produces other types of products, such as ammonia, branched-chain fatty acids (BCFAs), and other undesirable and potentially toxic molecules (e.g., phenolic compounds and some biogenic amines), that possess proinflammatory action [12,13,14].

The most used prebiotics in human and animal nutrition belong to the oligosaccharide family, which includes MOSs, which have several biological effects. MOSs prevent dental caries, reduce the serum levels of total cholesterol and lipids, and increase beneficial gut bacteria growth in the gastrointestinal tract. These oligosaccharides are not digested in the small intestine; once they reach the large intestine, they are fermented by anaerobic bacteria, producing a large number of volatile fatty acids (VFAs) and CO_2_, ammonia, and H_2_ [15]. Besides MOSs, the yeast cell wall also contains beta-glucans with prebiotic and immunomodulatory functions.

The pet microbiome is important to owners as they share the same home. Indeed, a possible exchange of microorganisms between pets and their owners has been previously reported [16]. Furthermore, pets may suffer from several medical conditions, such as obesity, cardiovascular diseases, diabetes, and periodontal disease; these conditions have been previously reported to be microbiota-related in humans [17]. Studies on the pet microbiome also include disease conditions [18,19,20]; however, they also assess the effects of dietary macronutrients and fibers [21,22].

A metataxonomic 16S rDNA analysis allowed us to correlate the changes in the abundance of specific bacterial taxa with functional properties. However, the estimates were often not precise due to a variation in functionality that could be found within the genera [23]. Thus, new approaches to predict the microbiota metabolic behavior using gene and genome databases have been widely used [19,21,24,25].

Specific protocols to assess intestinal fermentation are used in nutrition trials [26]. In many studies, animals are kept in metabolic cages to ensure the best results via fresh feces sampling and food intake recording [27]. However, despite being a noninvasive procedure, housing animals in metabolic cages is stressful. Studies with dogs have shown that single accommodations negatively influence their behavior, leading to abnormal movements, a decreased sleep time, and an increased vocalization rate [28]. Metabolic cages typically contain no or limited environmental enrichment to avoid its interference with the organic compounds to be tested [29].

Nowadays, animal welfare is a significant concern. Hence, initiatives are needed to explore and introduce new methodologies that may benefit animals without compromising the scientific results and to even improve the quality of scientific data [30]. Thus, developing techniques that provide the same results with free animals or even using in vitro methods that simulate the in vivo experimental conditions is of paramount importance to the future of science [30].

One widely explored tool that could replace animals in experiments is in vitro digestion and fermentation techniques. In vitro fermentation has been studied in many animal species [30]. Different organic materials (feces; rumen fluid; and ileum, cecum, and colon content) are used as inoculums to provide microorganisms (mainly bacteria) for the in vitro technique. When different substrates are provided, microorganisms produce a wide range of compounds that are useful for measuring the parameters and final products of fermentation at varying times in a controlled environment [31,32]. In vitro fermentation allows one to measure essential parameters, such as substrate disappearance, incubation residues, new products originated from fermentation, microbial biomass, gas volume, SCFAs, BCFAs, and potentially toxic products [33].

However, studies that compare in vitro and in vivo fermentation are scarce. A previous study with dogs and cats [34,35] reported that in vitro experiments could predict the in vivo use of fibrous compounds, which are essential to balance diets. Studies performed with pigs have also shown that in vitro techniques can predict the in vivo behavior of substrates, especially oligosaccharides [36]. Another study [37] was performed to compare different dietary substrates in goats that were offered based on rumen size. The authors reported that only one fermentation product was similar between the in vitro and in vivo experiments. Although the search for methods to replace the use of animals in experiments is of paramount importance, the results of in vitro studies compared to those in vivo seem to be conflicting. Another important aspect is that the fecal score can only be assessed in vivo.

Therefore, this study assessed the in vivo and in vitro fermentative responses and fecal microbiota in cats receiving diets supplemented with a blend of yeast cell wall compounds.

## 2. Material and Methods

### 2.1. Animals and Experimental Diets

The experiment was conducted at the Laboratório de Nutrição e Metabolismo de Felinos Domésticos (CEENUFEL) which belongs to the Departamento de Zootecnia (DZO) of Universidade Estadual de Maringá (UEM, Maringá-PR, Brazil). All animal and experimental procedures were approved by the local Ethics Committee on Animal Use in Experimentation (protocol no. 3158280121). 

Eighteen mongrel adult cats (nine male and nine female, 5.16 ± 0.85 years old and body weight of 4.35 ± 1.11 kg) were housed in individual metabolic cages with free access to fresh water. Diets were offered twice daily at the maintenance level throughout the experiment [26].

The study was designed as a randomized block over time with nine cats (*n* = 9) in each 27-day block (2 blocks), totaling eighteen cats (*n* = 18). Each block had three cats for each treatment, and three treatments had six cats (*n* = 6) each.

A commercially available adult maintenance cat food (kibble) was used as a negative control. Two levels (0.15% and 0.30%) of a commercial blend (Alltech Advantage Pet Biobalance FT, Alltech, Brazil) of mannan oligosaccharides (MOSs, 25 g/kg) and beta-glucans (BG, 5.8 g/kg) were top-dressed on food. The three treatments were as follows: (1) negative control group (Co) used commercial cat food coated with poultry fat (5%) and liquid flavoring (2%); (2) YCW-15 group used control food top-dressed with 0.15% of the commercial blend; and (3) YCW-30 group used control food top-dressed with 0.30% of the commercial blend. Poultry fat and liquid flavoring were included to improve food acceptance and to facilitate adherence to the commercial blend. The chemical composition of diets is found in Table 1.

Cats were adapted to diets for 14 days. Fecal samples were taken from d 15 to d 21 of each block to assess digestibility and fecal score. From d 16 to 22 and d 22 to 27, feces were sampled for fermentation products and microbiome analyses. 

Using these feces samples, three experiments were performed: Exp. 1—effects of yeast cell wall derivatives on digestibility, fermentation products, and microbiota in cats; Exp. 2—in vitro fermentative properties of yeast cell wall derivatives; Exp. 3—in vitro fermentative behavior of yeast cell wall derivatives in different substrates (pectin, amino acids, or cellulose).

### 2.2. Experiment 1—Effects of Yeast Cell Wall Derivatives on Digestibility, Fermentation Products, and Microbiota in Cats

#### 2.2.1. Apparent Digestibility Coefficients (ADC) and Fecal Traits

The apparent digestibility coefficient of nutrients and metabolizable energy (ME) of diets were determined via the total collection of feces and urine method [26]. The food offered and refusals were weighed at each meal to record daily food intake. Total produced feces were taken after a 24 h period for six days. Fecal samples were weighed, packed in individual plastic bags, and stored at −15 °C for subsequent analysis. On collection days, feces were scored by the same researcher (F. G.) on a 5-point scale as follows: 1 = pasty and unformed; 2 = soft, poorly formed stool; 3 = soft, formed, and moist stool; 4 = hard and well-formed stool; and 5 = hard, formed, and dry stool [38]. At the end of the collection period, samples were thawed and pooled by animal.

Feces samples were weighed, identified, and stored at −15 °C. Afterward, feces samples were dried at 55 °C for 72 h. Diets and feces samples were ground in a ball-type mill and then analyzed to determine chemical composition according to methods of AOAC [39]: dry matter (DM, method 935.29), crude protein (CP, method 954.01), crude fiber (FB, method 962.10), acid-hydrolyzed ether extract (AEE, method 954.02), ash (method 942.05), and organic matter (OM, determined as the percentage of the difference between DM and ash). The gross energy (GE) was determined in a bomb calorimeter (Parr Instrument Co., model 6200, Moline, IL, USA). The apparent digestibility coefficients of DM, OM, CP, AEE, GE, and ME of the diets were calculated considering the total feces collection procedures with no urine sampling [26].

#### 2.2.2. In Vivo Fermentation Products

Fresh feces (10 g) were collected by grab sampling from spontaneous defecation, were mixed with 30 mL of 16% formic acid, and were stored at 4 °C for 24 h until VFA analysis. Afterward, samples were centrifuged (Rotina 420R, Hettich Lab Technology, Tuttlingen, Germany) at 4000× *g* for 15 min at 15 °C (the supernatant was collected and further centrifuged twice). Then, an aliquot of the supernatant was placed into a 2 mL Eppendorf tube, was centrifuged at 14,000 rpm for 15 min at 4 °C, and was stored at −15 °C.

VFAs were determined via gas chromatography [40] in a Shimadzu chromatograph (GC- Plus 2010) equipped with a flame ionization detector (FID). Chromatographic conditions were set based on literature data and previous tests to obtain a greater sensitivity. The column used in the gas chromatograph was ZB-WAX-PLUS with a stationary phase (100% polyethylene glycol) with high polarity. The following variables were established: chromatographic column size (30 m × 0.32 mm) and film thickness (0.50 μm). Injector and detector temperatures were maintained at 250 °C. The column temperature was heated to 80 °C for 1 min and was then heated again to 235 °C at a rate of 35 °C min^−1^, and it was then kept constant for 5.00 min. The total analysis time was 10.29 min. Hydrogen (H_2_), with a continuous flow of 1.2 mL min-1, was used as carrier gas and nitrogen as make-up gas at 30 mL min^−1^. In the detector, the flame was produced using H_2_ and synthetic gas (40 and 400 mL min^−1^, respectively). Samples (1.0 µL) were injected using a 1:80 split ratio. VFAs were identified by comparing sample retention time with the analytical standard. Acetic, propionic, butyric, isobutyric, valeric, isovaleric, methylvaleric, hexanoic, and heptanoic acid were quantified.

Ammonia N (N-NH _3_) was assessed via distillation with KOH and subsequent titration. Briefly, samples (the same used for VFAs before the last centrifugation) were thawed at room temperature and diluted with distilled water (2:13, *v*/*v*). Then, a 2 mL aliquot was placed in a micro-Kjedahl tube, and 5.0 mL KOH (2N) was added. Samples were distilled at a 2 mL/min flow rate. The distillate was recovered in an Erlenmeyer flask containing 10.0 mL of boric acid (2%) until reaching a 50 mL volume and was then titrated with 0.005N HCl [41].

Briefly, 3 g of feces samples were diluted with 9 mL of distilled water and kept at 4 °C for 24 h. Then, samples were centrifuged at 4000 g for 15 min at 15 °C. This procedure was performed three times, and the final supernatant was stored at -15 °C. The lactic acid concentration was determined at 565 nm in a spectrophotometer using 0.1% lactic acid as standard as previously reported by Pryce [42]. Fecal pH was determined (K39—1014B, Kasvi, São José do Pinhais-PR, Brazil) in samples diluted 1:3 (*w*/*v*) with ultrapure water [43].

#### 2.2.3. Fecal Microbiota

Data on microbiota were obtained from 18 feces samples. Extraction of total DNA from feces samples was conducted using the Quick-DNA Fecal/Soil Microbe Miniprep Kit (Zymo Research) according to manufacturer’s instructions. PCR reactions were performed using a 20 μL mixture containing 10 μL of 2x GoTaq^®^ Green Master Mix (Promega, USL), 0.3 μM of a forward oligonucleotide, 0.3 μM of a reverse oligonucleotide, 2.2 μL of genomic DNA, and sterile ultrapure water to make up to 20 μL. Amplification involved an initial denaturation at 94 °C for 3 min followed by 30 denaturation cycles at 94 °C for 45 s, annealing at 55 °C for 1 min, extension at 72 °C for 1 min, and a final extension at 72 °C for 10 min. Amplification reactions were performed in a Veriti™ Thermal Cycler (Applied Biosystems). Amplification was confirmed via electrophoresis in 2% agarose gel stained with UniSafe Dye 0.03% (*v*/*v*) using ~ 400 bp (amplicon size). In this step, the indexers were inserted into standard adapters necessary for cluster generation and sample sequencing. The indexing reaction was performed following the Nextera XT Index Kit protocol (Illumina). The amplification program involved an incubation at 72 °C for 3 min and initial denaturation at 95 °C for 30 s followed by 12 cycles of 95 °C for 10 s, 55 °C for 30 s, and 72 °C for 30 s and final extension at 72 °C for 5 min. Amplification reactions were performed in a Veriti™ Thermal Cycler (Applied Biosystems). The generated libraries were submitted to purifying steps using magnetic bead Agencourt AMPure XP (Beckman Coulter) to remove small fragments and primer remnants. Real-time PCR was performed using Kapa-KK4824 Kit (Library Quantification Kit—Illumina/Universal) in a Quantstudio 3 (Applied Biosystems) following the manufacturer’s instructions. Before sequencing, all samples were normalized (3 nM) to generate an equimolar DNA pool. Sequencing was performed using a new generation Illumina MiSeq system (Illumina^®^ Sequencing) and a 300-cycle MiSeq Reagent Micro Kit (2 x 150 bp read length). Bioinformatics of the microbiome was performed using QIIME 2 2021.2. The raw sequence data were demultiplexed and filtered using the q2-demux plugin followed by noise removal with DADA2 (via q2-dada2). All amplicon sequence variants (ASVs) were aligned with math (via q2-alignment) and used to build a phylogeny with fasttree2 (via q2-phylogeny). Alpha diversity was determined via Faith’s phylogenetic diversity. Beta-diversity was analyzed using UniFrac, unweighted UniFrac, Jaccard distance, Bray–Curtis dissimilarity, and principal coordinate analysis (PCoA). They were estimated using q2-diversity after the samples were rarefied to 22,685 sequences per sample. Taxonomy was assigned to the amplicon sequence variant (ASV) q2 class-sklearn naïve Bayes taxonomy classifier against Greengenes operational taxonomic unit (OTU) 13_8 99%. Metabolic pathways were obtained via PICRUST2 software (2020) using taxa obtained in QIIME 2. First, a standardization based on the amount of 16S sequences per taxon was performed; then, pathways were numbered according to their presence and amount in each taxon. Metabolic pathways were compared via MetaCyc database to determine their products [44].

### 2.3. Experiment 2—In Vitro Fermentative Properties of Yeast Cell Wall Derivatives

#### 2.3.1. Experimental Design, Sampling, and Feces Transport

In this experiment, only fresh feces from animals in the control group (*n* = 6) of Exp.1 (adapted to the experimental diet) were used as fecal inoculum. Treatments YCW-15 and YCW-30 were formed by adding the respective prebiotic to the fecal inoculum. This experiment was performed with the same treatments as in Exp. 1 (in vivo) under in vitro conditions to reduce data variation and to isolate animal effects on fermentation products (all inoculums received the three treatments).

From each cat in the control group of Exp. 1, five grams of fresh feces were aseptically sampled and placed in 15 mL sterile Falcon tubes containing carbon dioxide (CO_2_). All samples were transported to the laboratory (within 3 h) at 39 °C to be used as inoculum [45].

#### 2.3.2. Fermentation Procedure

Feces samples were diluted 1:10 (*w*/*v*) with an aerobic sterile saline solution (9 g/L NaCl) at 39 °C and were vortexed for 60 s. The sterile condition was ensured via CO_2_ injection. After that, samples were filtered through four layers of sterile gauze (16 threads per cm^2^). Filtered samples were used as fecal inoculum in the fermentation bottles [46]. A 5-mL aliquot of the inoculum was added to a 120 mL bottle containing 82 mL of the modified fermentation medium [47]. Trace mineral solution was exchanged for 0.14% of a trace metal mix A5 with Co (Sigma–Aldrich) for each liter of basal solution. An iso ingredient mix (600 mg) containing 200 mg of an amino acid blend (BioTech Solutions, Suzhou, China), 200 mg of pectin (pectin, Zio Chemical, Guangzhou, China), and 200 mg of cellulose (cellulose, Zio Chemical, Guangzhou, China) was used as substrate. The three experimental treatments were constituted as in Exp. 1: (1) control used only inoculum and substrate; (2) YCW-15 group used control treatment added to 5 mg of the commercial product; and (3) YCW-30 group used control treatment added to 10 mg of the commercial product.

All fermentation vials were prepared one day before inoculation and kept at 4 °C for 12 h for substrate hydration [48]. Before inoculation, vials were kept at 39 °C for 1 h in an oven. After inoculation, vials were bubbled for 5 min with CO_2_, sealed with an aluminum seal and rubber stopper, and kept at 39 °C for 24 h. Each inoculum was analyzed in duplicate. Two vials with no inoculum or substrate were used as the negative control. After 24 h, the total gas production was measured with a pressure transducer (PXM409-3.5 BGUSBH, Omega, Connecticut, USA). This device was hermetically coupled with a hypodermic needle (18 G 1.60 × 40), transposing the rubber stopper and accessing the headspace. Pressure measured as PSI was transformed to volume (mL) using the following equation:

y = 3.7011x − 3.5724 (1)

where y = gas volume at 39 °C and x = pressure of gas phase (PSI) (R² = 0.9923).

The standard curve was built using six different fermentation vials containing 87 mL of distilled water (total volume of the inoculums). Known increasing amounts of air were applied to the gas phase in vials, and then they were kept at 39 °C for three hours. After that, the pressure was measured as PSI using the transducer.

In the liquid phase, pH was measured using a digital pH meter (AK90, Asko, Rio Grande do Sul, Brazil). Fermentation was stopped through cooling at 4 °C [49]. The liquid phase was sampled to perform VFA and ammonia analyses. For VFA analysis, samples were collected and centrifuged as described in Exp.1. Ammonia was analyzed as previously described by Houdijk [50].

#### 2.3.3. Analyses of Fermentation Products

For VFA and BCFA determination, 10 mL of each sample (liquid phase) was mixed with 30 mL of a 16% formic acid solution (1:3, *v*/*v*) and was kept at 4 °C for 72 h for precipitation. Then, samples were centrifuged three times at 4000× *g* for 15 min at 15 °C (Rotina 420R (Hettich Lab Technology, Tuttlingen, Germany)). Another centrifugation at 14000 rpm for 1 h at 4 °C was performed. The supernatant was sampled and stored at −20 °C [40] until chromatographic analysis as described in Exp. 1. Ammonia N was determined in the same centrifuged sample used for fatty acids after being thawed at room temperature and then diluted 2:13 (*v*/*v*) with distilled water (2:13, *v*/*v*). Ammonia N was determined via spectrophotometry [50].

### 2.4. Experiment 3—In Vitro Fermentative Behavior of Yeast Cell Wall Derivatives in Different Substrates

#### Experimental Design, Sampling, and Feces Transport

Feces from Exp. 1 (18 animals) were collected from d 22 to 27. These feces were submitted to in vitro fermentation as described for Exp. 2. However, only 200 mg of one of the following substrates was used: (1) amino acid blend (BioTech Solutions, Suzhou, China), (2) pectin (pectin, Zio Chemical, Guangzhou, China), and (3) cellulose (cellulose, Zio Chemical, Guangzhou, China). The main objective of using this design was to assess the behavior of the fecal inoculum of cats that received in vivo treatments when submitted to different fermentation stimuli. A negative control food with no substrate added to the inoculum was used. The analyzed chemical composition of the fermentative substrates is found in Table 2.

This study was designed within a 3 × 4 factorial arrangement of treatments with three in vivo treatments (feces of cats fed as control, YCW-15, and YCW-30) and four fermentative substrates (negative control, pectin, amino acids, and cellulose). All analyses were performed in duplicate. The other procedures in this study were identical to Exp. 2.

### 2.5. Statistical Analysis

Data on fecal score, apparent digestibility coefficient, and fecal production were submitted to variance analysis, and means were compared via Tukey test. Descriptive statistics and univariable screening were used for data on pH, gas volume, and fermentation products. Treatment effects were analyzed via MANOVA and Roy contrast (JMP versions 13.1–14.2, SAS Institute Inc., Cary, NC, 1989–2019), generating a biplot response graph. In Exp. 1, the gas volume was not analyzed. Microbiota diversity in alpha diversity was analyzed via Kruskal–Wallis test. Beta diversity was analyzed via PERMANOVA. Data on taxonomy was analyzed via chi-square test and underwent Bonferroni correction. Data on metabolic pathways were analyzed via Kruskal–Wallis test. In Exp. 3, treatments were analyzed in a block using substrates as independent variables.

Significant differences were set at *p* < 0.05, and trends were set at 0.05 ≤ *p* ≤ 0.10 except for taxonomy data, which were considered statistically different only when they had a *p* < 0.05 after Bonferroni correction.

## 3. Results

### 3.1. Experiment 1

The treatments did not affect (*p* > 0.05) the food intake of the cats. The average food intake of the cats fed the control, YWC-15, and YWC-30 were 52.71 ± 3.37, 57.72 ± 5.58, and 53.74 ± 3.93 g/day, respectively.

Cats fed YCW-30 resulted in greater apparent digestibility coefficients of CP, OM, and GE as well as the highest ME and fecal score (Table 3).

**Figure 1:**
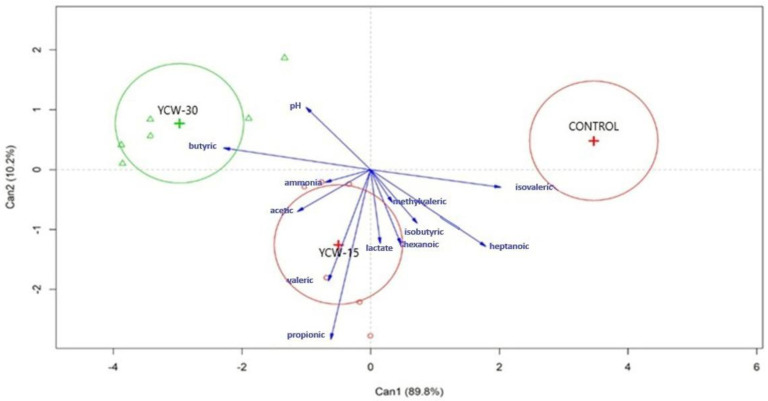
Biplot of canonical component analysis using the first and second components (PC1, 89.8%; PC2, 10.2%). Treatments are located according to their coordinates in the components. Vectors are related to the trend of the variables in the treatments.

The descriptive data of the fecal pH and the fermentation products are presented in Table 4. The treatment effects were assessed for these data via a multivariate analysis (Table 5 and Figure 1).

No treatment effect (*p* > 0.10) was observed for the fecal pH or the fermentation products (Table 4). Figure 1 shows the vectors of the canonical correlation analysis. Components 1 and 2 explained all the variations of the results. All the fermentation markers (pH, SCFAs, and ammonia) were highly correlated with YCW (0.15% or 0.30%) except isovaleric, heptanoic, and methylvaleric acid, which were most influenced by the control treatment.

The proximity and longitude of the vectors within each group express the relationships among the groups and variables. The correlation among the variables is represented by the angle formed among their vectors. Propionic, valeric, acetic, lactic, hexanoic, 4 methylvaleric, and isobutyric acid were the most affected by YCW-15. Butyric acid and the pH were more affected by YCW-30. Ammonia showed a low correlation with the treatments. However, a trend in the cats fed YCW-15 and YCW-30 was observed.

**Figure 2:**
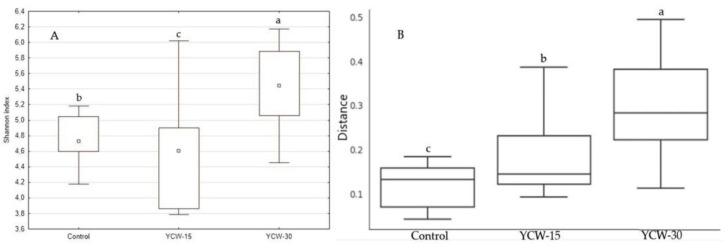
Microbiome alpha diversity (**A**) and beta diversity (**B**) of control, YCW-15, and YCW-30 groups. ^a,b,c^ Boxplots with the same letter are similar.

Shannon’s alpha diversity index (Figure 2A) showed differences (*p* < 0.001) among the treatments. The highest index was observed in the YWC-30 group (YCW-30 = 5.44; YCW-15 = 4.60, and control = 4.73). The fecal microbiota of the cats fed YCW-30 were different (*p* = 0.008) from those fed the control as suggested by beta diversity (Figure 2B).

**Figure 3:**
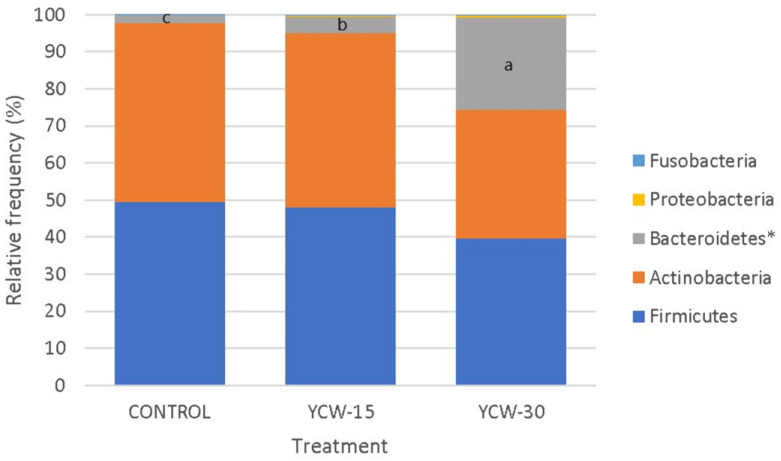
Relative frequency variation of the main fecal bacterial phyla in control, YCW-15, and YCW-30 groups. ^a,b,c^ Boxes with the same letters are similar at *p* ≤ 0.003. * Phylum with significant differences.

**Figure 4:**
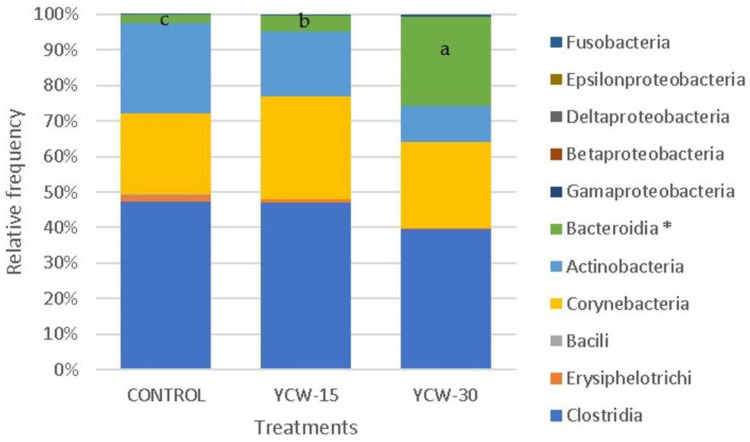
Relative frequency variation of the main fecal bacterial classes in control, YCW-15, and YCW-30 groups. ^a,b,c^ Boxes with the same letters are similar at *p* ≤ 0.0015. * Classes with significant differences.

**Figure 5:**
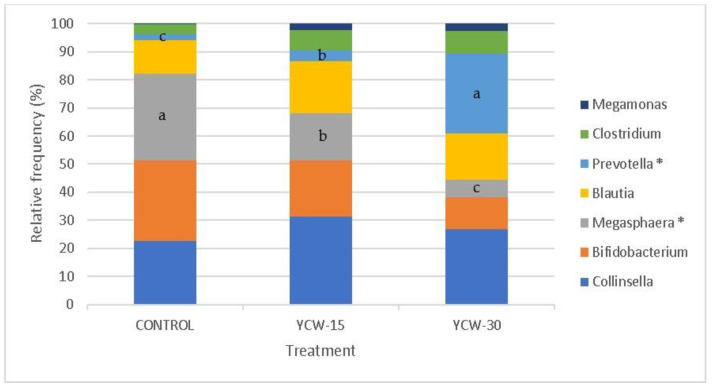
Relative frequency variation of the main fecal bacterial genera in control, YCW-15, and YCW-30 groups. ^a,b,c^ Boxes with the same letters are similar at *p* ≤ 0.00238i. * Genera with significant differences.

The microbiota operational taxonomic units (OTUs) showed 8 phyla, 19 classes, 20 orders, 40 families, and 66 genera. The present study analyzed five phyla (Figure 3), representing 99.93% of the total abundance. Differences (*p* = 0.0001) among treatments were observed in the phylum Bacteroidetes (control = 2.23%, YCW-15 = 4.40%, and YCW-30 = 24.95%). A total of 11 classes (Figure 4) were analyzed (99.90% of total abundance). B. bacteroidia differed (*p* < 0.0001) among the treatments (control = 2.23%, YCW-15 = 4.40%, and YCW-30 = 24.95%). Regarding the genera, a total of seven (89% of abundance) were analyzed. Differences among treatments (*p* < 0.001) were observed for Prevotella (Co = 2.05%, YCW-15 = 3.93%, and YCW-30 = 28.21%) and Megasphaera (Co = 30.82%, YCW-15 = 16,74%, and YCW-30 = 6.30%) (Figure 5).

**Figure 6:**
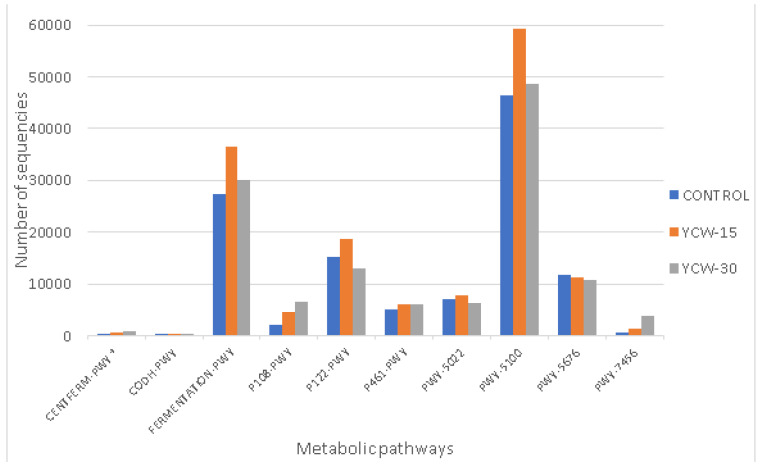
Variation of metabolic pathways of the main bacterial fermentation sequences of the 16S rRNA gene determined via MetaCyc. * Pathway with differences among treatments.

A total of 487 metabolic pathways of the 16S gene were found in the present study. However, only ten pathways (3.23%) that were related to the fermentation products (Table 6) were used (3,094,291 sequences). Figure 6 summarizes the absolute results of the fermentation sequences in the pathways.

**Figure 7:**
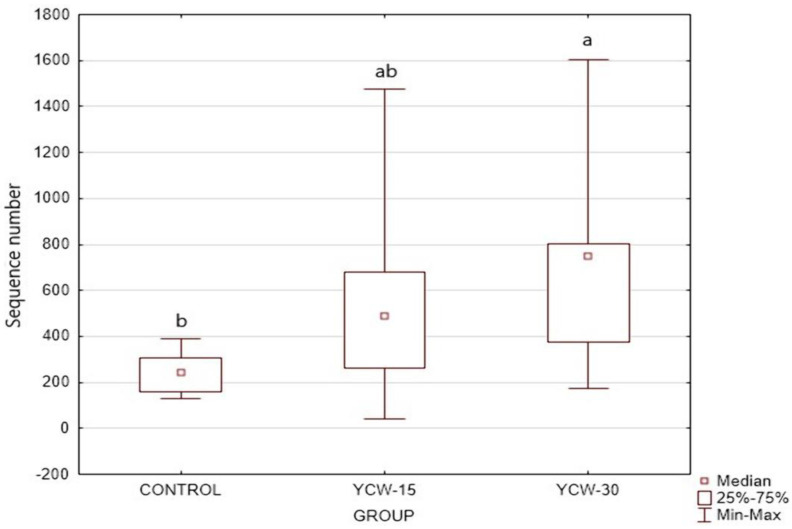
Median differences (absolute values) among treatments in the metabolic pathway CENTFERM-PWY. ^a,b^ Boxplots with the same letters are similar at *p* ≤ 0.1.

The CENTFERM-PWY pathway tended to be different (*p* = 0.086) in the YCW-30 group compared to the control (Figure 7).

### 3.2. Experiment 2

Table 7 shows the means and standard errors of the in vitro fermentative parameters of the fecal inoculum of the cats (fed the control food) with additives.

**Figure 8:**
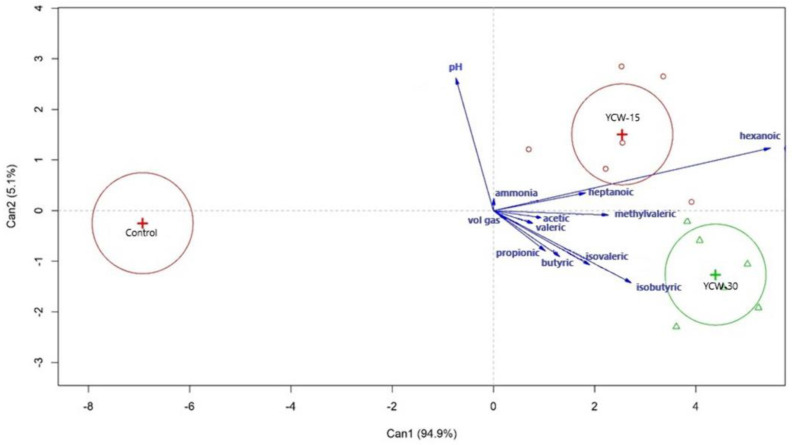
Principal component analysis biplot using first and second principal components (PC1, 94.9%; PC2, 5.1%). Treatments are located according to their coordinates in the components. Vectors relate the trends of variables to treatments.

The treatment effects (Table 8 and Figure 8) were assessed for these data via a multivariate analysis, and it showed that the treatment effects were significant (*p* = 0.006).

A higher pH was observed in the control and YCW-15 groups. The treatments containing the YCW additives showed higher effects on all the other fermentation metabolites (Figure 8).

### 3.3. Experiment 3

The results of the mean and standard error values of the fermentation parameters for each substrate in the in vitro experiment are shown in Table S1.

An effect of the treatment and fermentative substrate was observed. However, the fermentative substrate effect was much more pronounced (Table 9).

**Figure 9:**
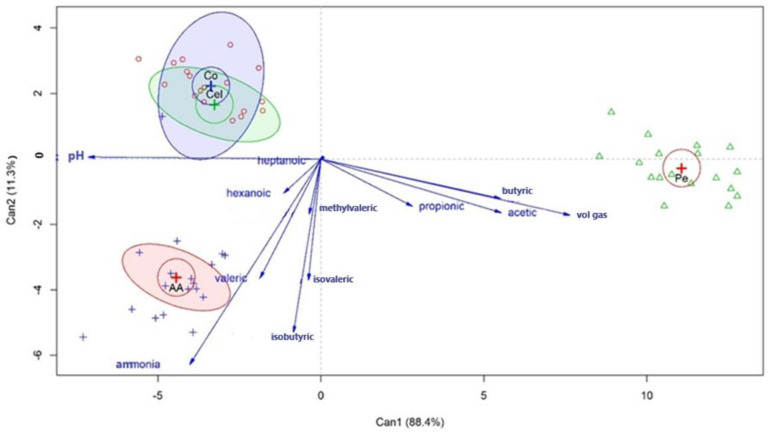
Biplot of principal component analysis using first and second principal components (PC1, 88.4%; PC2, 11.3%). Treatments are located according to their coordinates in the components. Vectors are related to the trend of variables in treatments.

The biplot of Exp. 3 was made using the results and was submitted to a principal component analysis so the variations of the fermentation products, gas volume, and pH among the substrates could be verified. The data were analyzed according to the groups in the components. Components 1 and 2 showed a total of 88.4% and 11.3% variance, respectively (Figure 9).

There was an increase in the pH of the inoculum in the control, cellulose, and amino acids groups, but not in the pectin group, which was related to acetic, propionic, and butyric acid and the gas volume. The inoculum supplemented with amino acids was related to ammonia, isobutyric, valeric, and isovaleric acid. This treatment tended to be related to 4 methylvaleric and hexanoic acid. Heptanoic acid was not associated with any group.

## 4. Discussion

The present study simultaneously determined the fermentation products in vivo and in vitro, aiming to verify the agreement of the data from both methods regarding the prebiotic effects of yeast cell wall derivatives and their impact on intestinal health. In vitro techniques have been evaluated and developed for a few decades. However, they are not considered a substitute for in vivo studies despite being of scientific interest since in vivo studies are expensive, take longer, and involve ethical issues [51,52]. Thus, the in vivo method remains a reference method.

In vitro techniques enable one to obtain results in a shorter time and to gain information on fermentation products’ kinetics and the metabolites of nutrient degradation. Furthermore, in vitro techniques isolate the sources of variation (temperature, dilution, nutrient flow, and individual interferences). Thus, in vitro techniques also work as a complementary technique to in vivo techniques. For this reason, many studies with prebiotics are performed using these techniques [21,45,46,47,53,54,55,56,57,58].

In the present study, Exp. 2 and 3 were performed using the feces of cats not adapted [22,45,46,59] to or adapted [13,49,54,60] to dietary prebiotics as an inoculum. The results from Exp. 1 (in vivo) and 2 (in vitro) were not conflicting. According to a canonical correlation, the treatments containing YCWs showed the pHs and fermentation products of the YCW-15 and YCW-30 groups.

Although a trend was observed in vivo (Exp. 1), the effects of the prebiotics on the in vitro fermentation products were more pronounced. This effect was supported by the intensity of the vectors and the treatment effects, which were all in the quadrants of the YCW-15 and YCW-30 groups. A less marked effect in vivo with more dispersed vectors was observed. In Exp. 3, although the prebiotic effect was significant, it was hidden by the fermentation substrate as suggested by the canonical correlation graph. The fermentation substrates showed an even more pronounced effect on the fermentation products as suggested by the direction and intensity of the vectors. Possibly, this occurred due to the low dietary levels of the prebiotics employed, which could have masked their effects on the effects of the substrates.

The effects of oligosaccharides were studied in cats fed higher concentrations than those employed in the present study. For example, 0.5% fructooligosaccharides (FOSs) and galactooligosaccharides (GOSs) [7] as well as 4% FOS and guar gum were used in the studies of Barry et al. [21] and Rochus et al. [61], and up to 9% FOSs were used in the study of Hesta et al. [62]. 

The oligosaccharides that are most evaluated in dogs and cats are inulin and FOSs. Dietary levels in studies range from 0.2% to 6% and from 0.18 to 9% [22,62,63,64,65,66,67]. The yeast cell wall contains three main groups of polysaccharides: mannose polymers (soluble mannan bound to proteins, about 40% of the cell wall DM), glucose polymers (b-glucan, about 60% of the cell wall DM), and the polymers of N-acetylglucosamine (chitin, about 2% of the cell wall DM) [68,69,70]. The dietary level of the YCWs (MOSs + b-glucans) in the present study was up to 92.4 mg/kg of the food, a much lower level compared to previous studies with dietary levels ranging from 2000 [71,72,73] to 14,000 mg/kg [63] of the food. The variables that we analyzed were already widely assessed in previous studies that determined the fermentation products using conventional statistical methods [4,34,65,74,75]. Alternative statistical models, such as multivariate analyses, are not usually applied in these studies. The benefit of this statistical approach is the simultaneous assessment of the effects of the parameters in an integrated way as they occur in the physiological systems, which is not necessarily observed in the univariate statistical approach [76]. We saw it very clearly when comparing the in vivo and in vitro data.

Although prebiotics influence fermentation in the large intestine, these substances can affect nutrient digestibility due to their solubility and viscosity. Additionally, the total apparent digestibility results from a balance of the nutrients ingested and excreted, and the disappearance or biotransformation of the nutrients in the large intestine is not considered [77]. Due to this low specificity of the total apparent digestibility, the results among studies on prebiotics are very variable.

We observed a greater digestibility of the protein, ME, and OM in the cats fed YCW-30. Similar results were reported by Middelbos et al. [73] and Barry et al. [66], who studied the effects of the dietary beet pulp (2.5%) and inulin (0.4%), respectively. However, other studies showed reduced digestibility coefficients of diets supplemented with prebiotics. For example, Patra [78] reported that the CP apparent digestibility in dogs tended to decrease when the dietary doses of the prebiotics (FOS, MOS, inulin) were greater than 2.82%. Propst [65] reported a lower CP digestibility in dogs fed dietary FOSs and inulin (dietary dose ranging from 0.3 to 0.9%). Middelbos [73] observed a reduction in the CP digestibility when dogs were supplemented with FOSs and yeast cell wall compounds (1.5%).

Prebiotics generally reduce the intestinal pH, leading to lower ammonia absorption in the colon and lower excretion in feces [79]. In addition, prebiotics can increase intestinal microbial diversity and, hence, microbial protein synthesis. This effect can lead to higher N fecal excretion, which is expressed as CP [80]. These two effects can be added to increase the intestinal viscosity, which can impair the action of the proteases in the small intestine and, hence, the digestibility of the protein and OM [2]. Although different from what was expected, the results that we observed in the present study have been reported in other studies [55,62].

We observed that the fecal score tended to be increased in the treatments containing the yeast cell wall derivatives. Prebiotic compounds promoted intestinal fermentation. They increased the osmolarity of the intestinal content and, hence, the moisture of the feces [2,73]. A feces quality reduction (low fecal score) was observed in some studies with dogs [64] and cats [2] that were fed FOSs. However, the dietary dose ranged from 0.17% to 6.17%, which are much higher concentrations than those that we used in the present study. Although we observed a trend towards a greater fecal score, all the treatments presented a score ranging from 3.67 to 3.87, suggesting that this effect depended on a low variation of the data.

Ammonia is a potentially toxic compound generated during the fermentation of nitrogen compounds in the large intestine [75]. A greater concentration of fecal ammonia may result from more colon microorganisms and, hence, increased urea hydrolysis to NH_3_ and NH_4_ [80] after supplementation with prebiotics [71,81]. However, this increased fecal ammonia concentration disagrees with other studies, where no effect on the fecal ammonia concentration was observed in dogs supplemented with FOSs [4,66,82] and FOSs and/or galactooligosaccharides [7]. That the data on the fecal ammonia concentration is conflicting may be due to factors other than prebiotic supplementation. Indeed, in addition to production, the fecal ammonia concentration is also affected by intestinal absorption (pH dependent) and the rate of its use in bacterial protein synthesis. Thus, the different metabolic pathways involving ammonia could help to explain the conflicting results among these studies.

Furthermore, ammonia can also be affected by experimental conditions such as the diet composition, digestibility, intestinal pH, and feces sampling and preparation [81]. The complex ammonia intestinal turnover could be demonstrated by the differences in the ammonia concentrations we observed between Exp. 1 and 2. In Exp. 1, the fecal ammonia concentrations were about 100-fold lower than those observed in the fermentation medium in Exp. 2. It is worth mentioning that, in Exp. 2, the total ammonia produced in a 24 h period was kept in a container until analysis. On the other hand, in Exp. 1, ammonia was measured directly in the feces sampled immediately after defecation. Even so, these concentrations represent the balance between the produced and the absorbed ammonia in the intestine or even that which was lost through volatilization.

Regarding the fecal pH, prebiotics can reduce proteolysis and the production of putrefactive intestinal compounds and can increase the number of beneficial bacteria. Thus, SCFAs are produced more abundantly, and the luminal pH decreases [83]. This expected pH reduction was evident in our study, likely due to the low concentrations of the prebiotics employed. However, the vectors showed a more significant interference of the treatments containing YCWs in the fecal pH and fermentation products. This finding was corroborated by the increase in the SCFA concentrations that we observed via a descriptive analysis. The less evident differences in Exp. 1 and the more dispersed vectors in this study could be explained by the physiological differences in the fermentation process in the large intestine. The pH is more acidic in the proximal colon due to SCFA synthesis during the fermentation of carbohydrates. However, in the more distal region of the colon, there are fewer fermentable carbohydrates. Therefore, the pH increases, and protein fermentation becomes more efficient [4]. Hence, ammonia production and the pH are increased.

On the other hand, in the in vitro batch experiments [21,84], as in the present study, there was no segmentation of the fermentation or absorption of the metabolites. These effects became more marked in Exp. 2 (in vitro) in which we observed higher amounts of SCFAs, BCFAs, 4-methylvaleric acid, hexanoic acid, and heptanoic acid in the treatments containing YCWs. These values were confirmed via MANOVA and vectors, all in the quadrants of the treatments containing YCWs, suggesting a strong influence of the treatments on these values jointly.

In experiment 2 (in vitro), the SCFA and BCFA increases were 9.46% and 9.26% in the treatments containing YCWs. Higher butyrate synthesis was desirable with supplementation with the prebiotics. It may be related to the increase of acetate-, lactic acid-, and formate-producing bacteria (*Bifidobacterium* and *Lactobacillus* spp.) that serve as precursors for the production of butyrate *by Eubacterium hallii* and *Anaerostipes caccae* [72].

Cats have a short digestive tract and a rapid intestinal transit. However, fermentation is also important for their intestinal health. Nutrients such as undigested starch and fiber are fermented, and VFAs are produced. They are important to intestinal epithelial cells’ growth, pH regulation, and immune function [85]. Santos et al. [72] supplemented adult cats with yeast cell wall derivatives (0.2% and 0.6%) and observed a greater fecal concentration of VFAs (up to 2.38 fold) when compared to the nonsupplemented control treatment. The authors observed an inverse relationship between SCFAs and lactate in this study. This was probably related to the fact that lactate is a precursor of VFA synthesis.

The greater VFA concentration that we observed was related to the dietary YCWs. In Exp. 1, only butyric acid was associated with YCW-30. In Exp. 2, marked VFA responses were observed for YCW-30. VFAs (acetic, propionic, and butyric acid) play an important role in glucose and cholesterol homeostasis. They serve as an energy source for colonocytes and the intestinal immune function. VFA production is related to carbohydrate fermentation. On the other hand, BCFAs (isobutyric, valeric, and isovaleric acid) are produced during nitrogen compound fermentation, which are considered undesirable fermentation products [26,43,86,87].

Flickinger et al. [82] observed that inulin (0.9%) promoted an increase in both SCFA classes, VFAs and BCFAs, in dogs. In another study, Beloshapka et al. [88] reported the positive (*p* < 0.05) effects of inulin and YCWs (1.4%) in diets for dogs on the production of VFAs with no accompanying change in BCFAs. Santos et al. [72] fed cats up to 0.06% YCWs and observed a linear increase in butyric and valeric acid. Similarly, Kanakupt et al. [7] reported that FOSs + GOSs (1% total) increased butyric and valeric (*p* = 0.07) acid and tended to increase the total SCFA concentration. This increase in SCFAs, especially in the butyrate concentration, was expected in the present study, as previous studies have reported the fermentability of yeast cell wall derivatives.

Hexanoic, 4-methylvaleric, and heptanoic acid have not been studied in pets. They have most commonly been reported in ruminant in vitro fermentation studies with strictly anaerobic bacteria (*Clostridium* spp. and *Megaesphaera* spp.) [89,90]. Hexanoic and heptanoic acid are medium-chain fatty acids common to silage or ruminal fermentation [13,91], and SCFAs are the substrates used for their synthesis. However, their synthesis is limited due to their toxicity to microorganisms [92]. In the present study, the level of these fatty acids in the in vivo experiment was approximately 100-fold lower than their concentrations in the in vitro experiment. Their proportion relative to the total fatty acids was also lower (5% of the total in Exp. 1 versus 11% in Exp. 2). These differences could possibly reflect the absorption of these compounds in the intestine as they are highly liposoluble.

The volume of in the vitro gas was used to measure the fermentation’s intensity, and there are different methodologies to do this [21,93]. Gas production [50,94] mainly correlates to carbohydrate fermentation to produce SCFAs and CO_2_. To a lesser extent, this is related to protein fermentation without any relation to fat fermentation. The higher production that we observed was associated with the YCWs with a higher trend towards YCW-30 in Exp. 2. In Exp. 3, it was related to pectin. These results were expected since fermentable carbohydrates were included. Barry et al. [22] fed pectin (4%) to adapted cats and reported higher gas production after 12 h of incubation using 150 mg of different substrates. Calabrò et al. [59], using the fecal inoculum of dogs, evaluated 500 mg of four different types of yeast cell wall compounds (as substrate) during a 72 h incubation and observed differences in three of them (*p* < 0.01). Bosch et al. [54] used eight fiber sources as the substrate (500 mg) and observed, in dogs, higher gas productions (*p* < 0.05) when using pectin, fructans, and gums (242.6, 257.3, and 251 mL of gas/g of OM, respectively) after 72 h of incubation. Rymer et al. [94] reported that data on gas production and its composition are difficult to compare among laboratories. Indeed, several factors, such as feces donors, previous diets, culture mediums, fermentation times, the type and amount of substrates, and the methodology, might interfere with the results.

The in vitro method showed a coherent response with the substrates. The direction of the vectors was related to the substrate. A greater SCFA production and gas volume and a lower pH were the expected results in pectin fermentation. This substrate was highly fermentable and soluble and was an important precursor of acetic, propionic, and butyric acid, which reduce the intestinal or inoculum pH [2,22,45,54,95]. On the other hand, the ammonia and BCFA vectors were, as expected, directed to the fermentation of amino acids. They were synthesized via amino acid deamination by proteolytic bacteria and increased the pH. We observed this effect, and previous studies had reported it [12,22,45,87]. Cellulose is a fiber resistant to fermentation in the large intestine of dogs and cats. As expected, cellulose fermentation yielded a low amount of the fermentation products from proteins and carbohydrates. A similar result was observed in other studies [22,25,26,96,97].

Although different sequencing technology has been used in previous studies with feline microbiota [30,63,98,99], the main reported phyla (Actinobacteria, Bacteroidetes, Fusobacteria, Firmicutes, and Proteobacteria) were similar to those we observed. In the present study, the phylum Firmicutes was predominant in all the treatments (mean = 45.71%), which agreed with previous studies [100,101]. The phylum Bacteroidetes and the Bacteroidia class showed the same proportions (10.53%), variations, and directions within the treatment. This finding showed the importance of the class in phylum conformation. These two taxonomic levels were significantly higher in the groups supplemented with YCWs. The phylum Bacteroidetes showed a higher proportion in the YCW-30 treatment (24.95%) compared to the other treatments and compared to the data from previous studies [5,100,102] that ranged from 0.45% to 4.6%. Our results suggested that different YCW concentrations selectively regulated the phylum and class of the intestinal bacteria. A similar effect was observed in a study [103] with piglets in which dietary insoluble and soluble fibers (cellulose 1% and 1% inulin, respectively) increased the phylum Bacteroidetes. In another study with cats [33] which used different dietary fiber sources (up to 4%), the phylum Bacteroidetes showed proportions of 40%, 33%, and 37% (cellulose, pectin, and FOSs, respectively) when microbiota was analyzed via DNA pyrosequencing. In a previous study [104], using the 16S rRNA method, authors suggested that the abundance of Bacteroidetes may be linked to the dietary fiber intake. In another study with five cats from different locations that were fed different diets [105], the authors analyzed the fecal microbiota via DNA pyrosequencing. They reported that Bacteroidetes/chlorobi was the predominant phylum (68%) without correlation with the fiber level. However, the relationship between Bacteroidetes and the soluble and insoluble fibers is still unclear [104]. Using 16S rRNA amplicon sequencing to identify taxonomy at the genus level has been questioned due to its low-resolution power [106]. The reasons for the variation are difficult to identify and are not obvious. Bias in the clone selection, primers, and sequencing depth may contribute to this divergence. Furthermore, different regions of the 16S rRNA gene reveal different diversity; hence, a given region may serve well to profile a certain spectrum of bacteria but not all. Although a microbial diversity analysis using pyrosequencing based on the 16S rRNA gene is a more economical method, a metagenomic approach to DNA is more ideal for characterizing bacterial diversity at the phylum level [105].

Despite the large number of taxa we observed, we analyzed 89% of the total using only seven genera. Higher proportions of Prevotella were reached in the treatments supplemented with YCWs. The highest proportion was observed in the YCW-30 group (28%), contrasting with a significant increase in Megasphaera in the control (31%). Prevotella, an important genus in SCFA synthesis, provides nutrients, regulates the intestinal barrier and immune system, and protects the intestine from pathogenic communities [107,108]. Our findings agreed with those observed in weaned pigs [109]: supplementation with YCWs (0.05%) increased the Prevotella genus. This finding was corroborated by previous studies in which yeast and its derivatives altered the intestinal microbiota [110].

Contrary to our observations, in a study [111] performed with dogs supplemented with YCWs (0.3% and 0.6%), a reduction in the Prevotella genus was reported. An increase in Prevotella may have resulted from a constant high intake of fibers since high-fiber long-term feeding is associated with an increased abundance of Prevotella [107,112]. Fiber’s effects on Megasphaera have been poorly studied. Previous studies have reported that Megasphaera is directly related to hexanoic acid in cats fed commercial dry food compared to those fed raw diets [113]. A greater Megasphaera abundance was observed in kittens compared to three other age groups [114]. The control treatment reduced Megasphaera due to the inclusion of the yucca extract (*Yucca schidigera*) in the additive. In a previous in vitro study [115] in which the different genera of pure bacteria and the yucca extract were used, no growth in Megasphaera was reported, even at the lowest levels of the yucca extract (0.7 mg/mL). Other bacterial groups were not affected. This effect was explained by the antibacterial and antiprotozoal activity provided by saponins, suggesting that the yucca extract may also contain other components with antibacterial activity. 

Alpha and beta diversity analyses are the most common and most relevant statistics for metagenome studies, providing an accessible and visual determination of the groups’ presence among the samples [116]. The Shannon index and beta diversity suggested greater diversity in the YCW-30 treatment compared to the other treatments. In a study with hens [117], higher directions of alpha diversity were observed when hens were fed YCWs (0.02%). It was assumed that the YCWs could improve the intestine microbial population and structure, which may be related to intestinal homeostasis. In other studies that were performed with dogs (125, 250, and 500 mg YCW/d) [118], pigs (0.05% YCWs) [109], and humans using an in vitro model (0.4% YCWs) [119], no effect of YCWs was observed. Beta diversity graphically represents the distance among the microbiological communities of each sample [116]. In the present study, beta diversity reflected a significant difference in the intestinal microbiota between the YCW-30 group and the control. It showed positive relationships between the beta diversity of the YWC-30 group and the intestinal microbiota. For example, individuals fed with different dietary compositions also had different intestinal microbial communities. Studies on YCW supplementation have shown varied results regarding beta diversity. There are studies in which dissimilarity is absent [109,111,118] or present [117].

In our study, different metabolic pathways related to fermentation products were compared among the treatments using metagenomics. The pathway end products were the same as the fermentation products of the experiments. In addition to the differences in the composition of the microbiota along the GI tract, each animal has a unique microbial profile. In a human study [120] using whole metagenome sequencing data on 1004 twins, the authors reported that unrelated individuals shared, on average, almost twice the number of metabolic pathways (82%) than those within the species (43%). However, the metagenomes (i.e., functional gene content) were maintained, suggesting that the functional aspects of microbiomes are similar in individual animals [32].

In the genome, the pathways that encode microbial metabolite synthesis are often physically grouped in regions known as metabolic gene clusters, which can be a predictive tool in metabolomics [35,121]. We observed only ten metabolic pathways that could predict the fermentation products. Only one, CENTFERM-PWY, tended to be affected by the treatments, and the YCW-30 group tended to show a greater number of gene sequences than the control. Butyric acid was formed from pyruvate as the end product of these pathways. Pyruvate is derived from glycolysis using the ferroxidin pathway [44]. This finding corroborated the high butyric acid in the fermentation products that we observed. A predictive approach to bacterial fermentation products related to the microbiome genome in cats using the MetaCyc database was not found in the literature. However, there are approaches comparing sequences with the Kyoto Encyclopedia of Genes and Genomes (KEGG) [30,122,123,124]. The KEGG contains significantly more compounds than MetaCyc, while MetaCyc contains significantly more metabolic reactions and pathways than KEGG, whose modules are incomplete [125]. Although many studies focus on the effect of microbial taxa, their metabolic potential has been little explored.

The in vivo and in vitro experiments did not show comparable results, which were expressed as different absolute values with similar tendencies. However, the in vitro technique provided unique conditions, such as standardized fermentation conditions, no interference of absorption in the fermentation product measurements, and a fermentation kinetics assessment. This suggested that this method could help study the fermentation capacity of the intestinal microbiota in cats under controlled conditions with results that complement the in vivo findings. These advantages of the in vitro technique were observed in our study. The concentrations of the prebiotics we used were low, and the in vivo responses were minimized, indeed. However, they were explored further via the in vitro experiments due to a better isolation of the factors.

## 5. Conclusions

According to the findings in this study, we could conclude that using yeast cell wall compounds, even at low dietary concentrations, affects short-chain fatty acid production, alters the intestinal pH, and modulates the fecal microbiota in cats. These responses were more pronounced under in vitro conditions due to better experimental control regarding the environment, animals, and sampling.

Although the in vivo and in vitro effects on intestinal fermentation showed different results, expressed as absolute values, both methods can be used together to complement their results and confirm their findings. Using metagenomics as a predictor of the fermentation products seems to be a promising tool for understanding the metabolic behavior of the microbiota as a whole.

## Figures and Tables

**Table 1:** Chemical composition of experimental diets.

Item	Co	YCW-15	YCW-30
Moisture (%)	7.20	7.30	7.30
Crude protein (%)	28.6	28.7	28.7
Ether extract (%)	12.22	12.84	12.69
Crude fiber (%)	3.51	3.62	3.58
Ash (%)	9.50	9.60	9.40
Nitrogen-free extract (%)	39.2	38.9	39.1
Organic matter (%)	90.6	90.4	90.6
Gross energy (MJ/kg)	18.18	18.28	18.31

Ingredient composition: soybean meal, corn grain, broken corn, corn gluten meal, broken rice, beet pulp, poultry byproduct meal, flaxseed, salt, poultry oil, fish oil, zinc proteinate, sodium hexametaphosphate, colorants, flavoring, methionine, taurine, phosphoric acid, vitamin and mineral premix, antifungals, antioxidants, and acidifier. Co: negative control group YCW: yeast cell wall.

**Table 2:** Chemical composition of fermentation substrates used in vitro.

Item	Pectin	Amino Acids *	Cellulose	YCW **
Moisture (%)	9.2	0.4	4.26	5.4
Crude protein (%)	2.2	97.5	0.16	23.19
Ether extract (%)	0.3	0.2	0.3	0.2
Crude fiber (%)	9	0	93	3.3
Ash (%)	2.0	0	0	14.3
Nitrogen-free extract (%)	77.3	2.1	2.28	53.61
Organic matter (%)	98	100	100	85.7
Gross energy (MJ/kg)	13.93	19.07	16.11	14.91

* Contained (per 530 g) 113 g of L-leucine, 56 g of L-valine, 56 g of L-isoleucine, 30 g of L-tryptophan, 23 g of L-tyrosine, 100 g of L-arginine, 71 g of L-glycine, 23 g of L-alanine, 20 g of L-glutamine, 19 g of L-lysine, and 19 g of L-taurine. ** Contained mannan oligosaccharides (2.50%), beta-glucans (0.58%), yucca extract, calcium carbonate, zinc proteinate, silicon dioxide, and oregano essential oil.

**Table 3:** Apparent digestibility coefficients, metabolizable energy, and fecal score.

Item	Co	YWC-15	YWC-30	Mean	SEM +	*p* Value
Dry matter %	70.7	69.7	72.3	70.87	0.763	0.177
Crude protein %	77.8 ^b^	76.3 ^b^	80.8 ^a^	78.30	1.304	0.013
Ether extract %	81.9	81.2	83.4	82.15	0.672	0.268
Ash %	20.9	20.1	18.3	19.77	0.782	0.750
Crude fiber %	47.3	43.0	51.5	47.28	2.464	0.461
Nitrogen-free extract %	76.1	75.8	77.5	76.47	0.530	0.168
Organic matter %	76.6 ^b^	75.4 ^b^	78.4 ^a^	76.78	0.880	0.046
Gross energy %	77.5 ^b^	76.8 ^b^	79.7 ^a^	78.00	0.885	0.057
ME (MJ/g)	14.3 ^b^	14.4 ^b^	14.9 ^a^	14.5	0.039	0.064
Fecal score	3.7 ^b^	3.8 ^b^	3.9 ^a^	3.8	0.070	0.078
Fecal excretion (g/MS/day kg^0.67^	5.413	5.762	5.427	5.534	0.114	0.838

^+^ SEM = standard error of the mean. ^a,b^—means followed by similar letters do not differ according to Tukey test.

**Table 4:** Fecal pH and fermentation products (mmol/g fecal DM) in Exp. 1.

Item	Control	YCW-15	YCW-30	Mean	SEM *
Ammonia	130.17	136.88	138.28	135.11	3.54
Fecal pH	5.82	5.82	6.03	5.89	0.07
Lactate	3.59	4.56	3.18	3.77	0.41
Acetic acid	229.16	268.99	272.74	256.97	19.72
Propionic acid	60.42	82.44	64.05	68.97	9.64
Butyric acid	58.28	71.00	82.97	70.75	10.08
Valeric acid	17.25	21.05	18.44	18.91	1.59
Total SCFAs	365.11	443.45	438.21	415.59	43.80
Isobutyric acid	41.44	42.86	27.81	37.37	6.78
Isovaleric acid	31.21	27.74	24.58	27.84	2.71
Total BCFAs	72.65	70.6	52,39	65.21	11.15
Methylvaleric acid	5.836	6.079	5.022	5.65	0.45
Hexanoic acid	2.51	2.67	2.29	2.49	0.16
Heptanoic acid	17.49	17.17	15.67	16.78	0.80

* SEM = standard error of the mean.

**Table 5:** MANOVA results in Experiment 1.

	DF	Roy Test	F Value	DF Numerator	DF Denominator	*p* Value
Treatment	2	84.396	35.165	12	5	0.08726 *

* < 0.1 indicates trend to significance.

**Table 6:** Fermentation pathways related to bacterial fermentation products using the MetaCyc database.

Pathway	Role
CENTFERM-PWY	Butyrate synthesis from pyruvate
CODH-PWY	Acetate synthesis from AcetylCoA
FERMENTATION-PWY	Acetate and lactate synthesis from phosphoenolpyruvate
P108-PWY	Propionate synthesis from pyruvate
P122-PWY	Lactate synthesis from glucopyranose
P461-PWY	Acetate and lactate synthesis from phosphotransferases
PWY-5022	Acetate and butyrate synthesis from aminobutanate
PWY-5100	Acetate and lactate synthesis from pyruvate
PWY-5676	Butyrate synthesis from AcetylCoA
PWY-7456	Degradation of B 1,4 mannan oligosaccharides for glycolysis

**Table 7:** Fermentation products (mmol), gas volume (mL), and in vitro pH corrected for fecal dry matter using fecal inoculum from cats fed control diet in Exp. 2.

Item	Co	YCW-15	YCW-30	Mean	SEM *
Ammonia	6.646	6.707	6.615	6.656	0.027
pH	6.217	6.242	6.167	6.208	0.022
Gas volume	288.7	290.1	292.3	290.4	1.040
Acetic acid	618.9	668.4	687.0	658.1	20.34
Propionic acid	490.0	501.6	524.1	505.2	10.01
Butyric acid	285.3	297.0	315.6	299.3	8.846
Total SCFAs	1394	1466	1526	1462	38.35
Isobutyric acid	246.4	258.1	270.8	258.4	7.039
Isovaleric acid	246.7	258.4	272.3	259.1	7.394
Valeric acid	252.5	264.2	271.8	262.8	5.613
Total BCFAs	745.6	780.8	814.9	780.4	19.99
Methylvaleric acid	23.30	35.00	37.80	32.02	4.443
Hexanoic acid	12.99	23.51	23.20	19.90	3.458
Heptanoic acid	209.4	221.1	221.1	217.19	3.904

* SEM = standard error of the mean.

**Table 8:** MANOVA results in Exp. 2.

	DF	Roy Test	F Value	DF Numerator	DF Denominator	*p* Value
Treatment	2	29.499	12.291	12	5	0.006088 *

* < 0.05 indicates significant differences.

**Table 9:** Multivariate analysis of fermentation products of different substrates using feces from cats adapted to the additive.

	DF	Roy Test	F Value	DF Numerator	DF Denominator	*p* Value
Treatment	2	1.073	5.006	12	56	1.428 × 10^−5^ *
Substrate	3	44.780	212.707	12	57	< 2.2 × 10^−16^ *

DF = degrees of freedom. * < 0.05 indicates significance differences.

## Data Availability

Data sharing not applicable.

## Supplementary Materials

The following supporting information can be downloaded at https://www.mdpi.com/article/10.3390/ani13040637/s1, Table S1: Fermentation products (mmol), gas volume (mL), and pH of in vitro experiment corrected for fecal DM (g) using different substrates and feces of cats of the in vivo experiment as inoculum.
