# Supplementary material for: Yeast Cell Wall Compounds on The Formation of Fermentation Products and Fecal Microbiota in Cats: An In Vivo and In Vitro Approach
Source: Animals (Basel). 2023 Feb 11;13(4):637. doi: 10.3390/ani13040637 (PMC9951743; doi:10.3390/ani13040637)
Supplement: Supplementary file 1 [file animals-13-00637-s001.zip › animals-2102218-supplementary.pdf]

Table S1: Fermentation products (mmol). gas volume (mL). and pH of in vitro experiment corrected for fecal DM (g) using different substrates and feces of cats of the experiment in vivo as inoculum.

	**Blank**					**Pectin**					**Amino acids**					**Cellulose**				
**Parameter**	**Control**	**YWC-15**	**YCW-30**	**Mean**	**SEM**	**Control**	**YWC-15**	**YCW-30**	**Mean**	**SEM**	**Control**	**YWC-15**	**YCW-30**	**Mean**	**SEM**	**Control**	**YWC-15**	**YCW-30**	**Mean**	**SEM**
Ammonia	3.34	3.60	3.67	3.537	0.102	2.92	2.99	2.72	2.879	0.081	7.44	7.53	7.16	7.374	0.112	3.83	4.18	3.72	3.909	0.137
pH	6.41	6.38	6.37	6.386	0.012	6.11	6.09	6.08	6.094	0.007	6.43	6.39	6.38	6.403	0.015	6.40	6.35	6.38	6.375	0.014
Gas volume	78.81	88.82	66.30	77.977	6.515	254.35	277.07	246.47	259.299	9.172	102.02	113.50	89.07	101.530	7.058	74.36	87.97	65.28	75.870	6.595
Acetic	484.63	473.52	419.57	459.240	20.092	712.45	773.40	755.97	747.271	18.123	515.50	536.12	514.12	521.914	7.116	514.22	482.23	433.85	476.768	23.360
Propionic	506.20	471.33	417.44	464.990	25.820	533.66	512.72	570.56	538.982	16.909	520.99	472.60	468.49	487.358	16.858	470.92	452.36	409.55	444.277	18.173
Butiric	241.21	244.20	216.23	233.882	8.868	248.65	265.35	227.24	247.081	11.027	302.96	301.35	261.26	288.520	13.638	239.59	247.30	224.15	237.014	6.807
SCFA Total	1232.04	1189.06	1053.24	1158.11	53.89	1494.77	1551.46	1553.78	1533.33	19.30	1339.44	1310.07	1243.87	1297.79	28.26	1224.73	1181.90	1067.55	1158.06	46.91
Iso-butiric	226.86	229.64	214.20	223.564	4.751	269.96	285.75	274.83	276.848	4.669	228.22	242.66	223.43	231.436	5.780	228.54	235.59	217.46	227.196	5.275
Isovaleric	228.67	225.97	208.55	221.065	6.306	241.62	234.90	216.49	231.006	7.512	265.23	262.04	231.98	253.085	10.591	225.16	229.51	213.89	222.855	4.654
Valeric	236.88	231.99	217.55	228.806	5.804	230.03	228.75	213.43	224.070	5.332	301.92	302.96	268.47	291.118	11.328	230.64	234.55	231.95	232.381	1.149
BCFA Total	692.41	687.60	640.30	673.43	16.63	741.62	749.40	704.75	731.92	13.77	795.38	807.66	723.88	775.64	26.12	684.34	699.65	663.31	682.43	10.53
4 metilvaleric	31.24	27.97	29.58	29.595	0.946	34.28	28.61	31.01	31.298	1.644	37.10	36.72	33.73	35.850	1.066	30.85	30.29	29.98	30.374	0.256
Hexanoico	29.91	26.93	21.56	26.133	2.445	24.86	24.95	21.46	23.756	1.150	29.49	28.03	22.31	26.609	2.189	24.55	23.92	21.33	23.267	0.985
Heptanoic	222.96	224.13	205.42	217.503	6.052	219.51	223.05	204.72	215.759	5.615	220.97	224.89	204.04	216.635	6.400	220.51	223.02	203.80	215.777	6.034
